# Current evidence for the effectiveness of heated and humidified high flow nasal cannula supportive therapy in adult patients with respiratory failure

**DOI:** 10.1186/s13054-016-1263-z

**Published:** 2016-04-28

**Authors:** Oriol Roca, Gonzalo Hernández, Salvador Díaz-Lobato, José M. Carratalá, Rosa M. Gutiérrez, Joan R. Masclans, for the Spanish Multidisciplinary Group of High Flow Supportive Therapy in Adults (HiSpaFlow)

**Affiliations:** Critical Care Department, Vall d’Hebron University Hospital, Vall d’Hebron Research Institute, Barcelona, Spain; Ciber Enfermedades Respiratorias (Ciberes), Instituto de Salud Carlos III, Madrid, Spain; Critical Care Department, Virgen de la Salud Hospital, Toledo, Spain; Respiratory Medicine Department, Ramón y Cajal University Hospital, Madrid, Spain; Emergency Medicine Department, Alicante General Hospital, Alicante, Spain; Anesthesiology Department, De Cruces General Hospital, Bilbao, Spain; Critical Care Department, Del Mar University Hospital, IMIM (Medical Research del Mar Hospital Institute), Barcelona, Spain

**Keywords:** High flow nasal cannula, High flow oxygen therapy, Respiratory failure

## Abstract

High flow nasal cannula (HFNC) supportive therapy has emerged as a safe, useful therapy in patients with respiratory failure, improving oxygenation and comfort. Recently several clinical trials have analyzed the effectiveness of HFNC therapy in different clinical situations and have reported promising results. Here we review the current knowledge about HFNC therapy, from its mechanisms of action to its effects on outcomes in different clinical situations.

## Background

Oxygen therapy is the main supportive treatment in hypoxemic respiratory failure and has traditionally been delivered using nasal prongs or masks. However, the maximal flow rates that these devices can deliver are limited because of the insufficient heat and humidity provided to the gas administered. It is accepted that flows up to 15 L/min can be delivered using conventional nasal prongs or masks, but this flow is far lower than the inspiratory flow of a patient with acute respiratory failure (ARF). Therefore, room air dilutes the supplemental oxygen, resulting in a significant decrease in the fraction of the inspired oxygen (F_I_O_2_) that finally reaches the alveoli.

In recent years, new devices that deliver totally conditioned gas through a nasal cannula at very high flow (up to 60 L/min) have emerged as a safe and useful supportive therapy in many clinical situations. High flow nasal cannula (HFNC) supportive therapy is one such technique that exerts its potential benefits through a variety of mechanisms. Here, we present a clinical review of the current knowledge of HFNC, from its mechanisms of action to its clinical indications in adult patients, and suggest future areas for research.

In the literature this treatment strategy has been variously described as nasal high flow and high flow oxygen therapy, but we believe that the most appropriate term is heated and humidified HFNC supportive therapy. This term reflects the features that generate the technique’s clinical effects (i.e., the delivery of warm and humidified air at high flows through a nasal cannula). Several HFNC devices are commercially available, but the technique always involves the delivery of a totally conditioned (37 °C containing 44 mg H_2_O/L [100 % relative humidity]) gas admixture via a wide bore and soft nasal prong at up to 60 L/min, with a fraction of inspired oxygen ranging from 0.21 to 1. In this study we also review the main clinical effects of HFNC therapy and how they are generated.

## Clinical effects and mechanisms of action

### Oxygenation improvement

Several mechanisms are known to contribute to oxygenation improvement, which is one of the main potential benefits of using HFNC therapy. However, the exact contributions of each mechanism have not been conclusively established and these may, in fact, vary from one patient to another.

#### Lower dilution of the administered gas with room air

During normal and quiet breathing, the peak inspiratory flow rate is around 30–40 L/min. During heavy workloads, a situation comparable with ARF, mean flow rates rise to more than 70 L/min [1]. Therefore, the peak inspiratory flow rate of ARF patients exceeds the flow rates supplemented by conventional oxygen devices, leading to a dilution of the gas administered and reducing the amount of oxygen that finally reaches the alveoli. Although the degree of dilution with room air depends on the mismatch between the flow administered and the patient’s inspiratory flow, it is much lower with a HFNC than with conventional oxygen devices. Thus, with the aim of reducing the entrainment of room air, it seems reasonable to try to match the flow rate of the HFNC with the patient’s peak inspiratory flow in order to maintain the F_I_O_2_ constant [2].

#### Positive end expiratory pressure effect

Another significant mechanism is the generation of a certain degree of positive airway pressure due to the resistance generated by the continuous administration of a high flow of gas [3–7]. In healthy volunteers breathing with their mouths closed, this pressure level may reach 12 cm of H_2_O at a provided flow of 100 L/min [5, 7] and it reaches its maximum at the end of expiration [3]. In a population of healthy volunteers, expiratory pressures with the mouth closed were higher than those with the mouth open at any given flow rate [5, 7]. Similarly, in ICU patients breathing with their mouths open and scheduled for weaning from mechanical ventilation, mean tracheal pressures dropped to 2 cm of H_2_O [8]. Therefore, major differences in the airway pressure achieved may be observed depending on the amount of flow delivered (the higher the flow, the higher the pressure), the type of breathing (with a significantly lower pressure in mouth breathers), the moment of the respiratory cycle, and the degree of respiratory failure. Electroimpedance tomography has been used to measure changes in bioimpedance caused by different lung conditions, especially those related to regional ventilation and it has been shown that variations in end-expiratory lung impedance correlate with changes in end-expiratory lung volume [9]. It has also been shown that HFNC therapy increases end-expiratory lung impedance [5, 10, 11] and is strongly correlated with airway pressure [10].

#### Dead space wash-out

It has been hypothesized that the continuous administration of a very high flow of gas flushes the carbon dioxide (CO_2_) out of the upper respiratory airway, avoiding the re-inhalation of the previous exhaled gas [12]. Although dead space was not measured, a recent in vitro experimental study using a silicone nasal cavity model demonstrated that fresh gas administered by HFNC at 30 L/min flushes the dead space in the nasal cavity and increases the F_I_O_2_ [13]. A randomized, crossover, experimental study with 13 neonatal piglets with lung injury treated with continuous positive airway pressure (CPAP; minimal leak) or a HFNC with a high or low degree of leak around the nasal prongs showed no changes in PaCO_2_ (arterial pressure of carbon dioxide) or PaO_2_ (arterial pressure of oxygen) with increasing CPAP [14]. Under both HFNC leak conditions, however, PaCO_2_ decreased and PaO_2_ increased with increasing flow until saturation. Beyond this saturation point no greater effect was observed, suggesting that all dead space had been flushed out. Therefore, this effect of nasopharyngeal dead space washout was associated more with increases in flow rates than with increases in pressure. One should bear in mind that, compared with the masks used with conventional oxygen delivery devices, nasal prongs reduce dead space. Moreover, continuous high flows delivered via a HFNC make rebreathing highly unlikely and, even if it occurs, differences in cannula size are unlikely to have any significant effect.

#### Effects on metabolic cost of gas conditioning and respiratory mechanics

An adult breathing with a 500-ml tidal volume and a respiratory rate (RR) of 12 breaths/min may expend approximately 156 calories/min conditioning gas [12]. If the gas is totally conditioned prior to administration, this metabolic cost can be avoided and, if no concomitant changes in cardiac output occur, oxygenation may be indirectly improved by reducing this energy requirement [12]. Moreover, the delivery of warm, humidified gas has been associated with better tolerance and comfort [8, 15, 16]; it improves the mucociliary function [17, 18], increasing mucus clearance and preventing atelectasis formation. Other results suggest that it may also improve the inspiratory effort [19]. It has been hypothesized that the mechanisms associated with this effect may be the increase in alveolar recruitment and the decrease in airway resistance. In fact, while it has been shown that airway resistance increases during nasal breathing of room air at 24 °C in 12 healthy subjects, no changes were observed during inhalation of moist air [20]. These results suggest that delivery of humidified gas can contribute to the decrease in airway resistance during HFNC therapy. Second, as a HFNC provides a certain level of positive pressure, it counteracts the collapse of the nasopharynx which occurs during normal spontaneous breathing and reduces upper airway resistance [21].

### Effects on respiratory pattern

Another of the main effects of HFNC is a significant change in the respiratory pattern. Compared with conventional oxygen, during HFNC use a significant decrease in respiratory rate (RR) has been described with no changes in PaCO_2_ [16, 22–24], as well as reduced thoraco-abdominal asynchrony [23]. This change in RR without affecting PaCO_2_ may suggest that alveolar ventilation remains constant and, therefore, tidal volume should increase or dead space decrease.

### Hemodynamic effects

As we noted above, HFNC increases lung volume and generates a certain level of positive pressure. It seems plausible that these changes may induce some hemodynamic effects; this issue was investigated in ten non-decompensated heart failure patients for whom sequential echocardiographies were performed at baseline, when using a HFNC at 20 L/min and 40 L/min, and post-HFNC use [25]. Since right atrial pressure has been considered as a surrogate of right ventricular preload and is most commonly estimated by inferior vena cava (IVC) diameter and the presence of inspiratory collapse [26], and since changes in the IVC diameter have been used to determine preload responsiveness in positive pressure ventilated patients [27, 28], the assessment of preload was inferred by the measurement of the degree of inspiratory collapse of the IVC. In these patients, treatment with a HFNC at 20 L/min and 40 L/min was associated with mean attributable reductions in the IVC inspiratory collapse of 20 % and 53 % from baseline, respectively. These changes were reversible after HFNC withdrawal and no other changes in ventricular function were reported, suggesting that HFNC therapy may be a useful supportive therapy in patients with acute cardiogenic pulmonary edema (ACPE), especially in those patients who do not tolerate non-invasive ventilation (NIV) but remain hypoxemic with conventional oxygen devices [29]. A recent retrospective cohort study that included 75 patients with ARF reported that, after adjusting for other clinical variables, patients with cardiogenic pulmonary edema as the cause of ARF were more likely to avoid intubation [30]. However, whether HFNC therapy can be compared to NIV or CPAP in terms of outcomes in hypoxemic ACPE patients remains uncertain.

### Better comfort

In ARF patients, the HFNC is better tolerated and more comfortable than conventional oxygen devices [16, 23] and NIV [8, 31, 32]. The heated humidification, the correction of the hypoxemia, the increase in alveolar recruitment, and the fact that the nasal prongs allow trouble-free speaking and eating must play some role in the improvement in comfort perceived by the patient during HFNC therapy.

## Clinical applications

Given its mechanisms of action and the effects generated, HFNC therapy has some potential benefits in different clinical indications. The most relevant studies are presented in Table 1 [10, 16, 22–25, 29, 30, 33–55].

**Table 1:** Most relevant studies of HFNC therapy

	Reference	Design	Patients	Results
Acute respiratory failure	Roca et al. [33]	Retrospective cohort	37 lung transplant recipients readmitted to ICU due to ARF (40 episodes)	The absolute risk reduction for MV with HFNC therapy was 29.8 % and the NNT to prevent one intubation with HFNC was 3. Multivariate analysis showed that HFNC therapy was the only variable at ICU admission associated with a decreased risk of MV (odds ratio 0.11 [95 % CI 0.02–0.69]; *P* = 0.02)
	Frat et al. [34]	RCT	310 ARF patients randomly assigned to HFNC, COT, or NIV	The hazard ratio for death at 90 days was 2.01 (95 % CI 1.01–3.99) with COT vs HFNC (*P* = 0.046) and 2.50 (95 % CI 1.31–4.78) with NIV vs HFNC (*P* = 0.006). In the subgroup of patients with a PaO_2_/F_I_O_2_ ≤ 200 mmHg, the intubation rate was significantly lower in the HFNC group
	Sztrymf et al. [22].	Prospective cohort	38 ARF patients	HFNC was associated with an early reduction of the RR, HR, dyspnea score, supraclavicular retraction and thoracoabdominal asynchrony, and better oxygenation. Absence of a significant decrease in the RR, lower oxygenation and persistence of thoracoabdominal asynchrony after HFNC initiation were early indicators of HFNC failure
	Roca et al. [16].	Prospective cohort	20 ARF patients who first received humidified oxygen with a bubble humidifier and delivered via face mask for 30 min and then via HFNC with heated humidifier for another 30 min	The HFNC was associated with less dyspnea and mouth dryness, and was more comfortable. HFNC was associated with higher and lower RR with no differences in PaCO_2_
	Sztrymf et al. [22]	Prospective cohort	20 patients with persistence of ARF despite COT	HFNC was associated with better oxygenation and lower RR
	Rello et al. [24]	Retrospective cohort	35 patients with ARF due to H1N1v pneumonia	After 6 h of HFNC O(2) therapy, non-responders presented a lower PaO_2_/F_I_O_2_. All eight patients on vasopressors required intubation
	Lemiale et al. [51]	RCT	100 immunocompromised patients with ARF randomized to a 2-h trial of HFNC vs COT	No differences on NIV or invasive MV during the 2-h period were observed. No differences in secondary outcomes (RR, HR, comfort, dyspnea, and thirst) were observed
	Mokart et al. [52]	Retrospective propensity-score analysis	178 cancer patients admitted to the ICU due to severe ARF	HFNC-NIV was associated with more VFD and less septic shock occurrence. Mortality of patients treated with HFNC was 35 % vs 57 % for patients never treated with HFNC (*P* = 0.008)
	Hyun Cho et al. [30]	Retrospective cohort	75 patients with ARF admitted to the ICU	62.7 % of patients successfully avoided intubation. APACHE II, SOFA, cardiogenic pulmonary edema, and improvement in oxygenation within 24 h were predictors of HFNC success
	Gaunt et al. [50]	Retrospective cohort	145 ICU patients who received HFNC	Subjects with a greater length of time between ICU admission and first use of HFNC had longer ICU and hospital LOS, even after controlling for adverse events and mechanical ventilation
	Nagata et al. [49]	Retrospective cohort (two periods: before-after)	83 (before) vs 89 (after) ARF patients	In the after period, fewer patients needed MV (NIV or MV): 100 % (before) vs 63 % (after) (*P* ≤ 0.01)
	Jones et al. [48]	RCT	303 hypoxemic and tachypneic patients admitted to the ED (165 HFNC vs 138 COT)	5.5 % of HFNC patients vs 11.6 % of COT patients required MV within 24 h of admission (*P* = 0.053)
	Kang et al. [53]	Retrospective cohort	175 patients who failed on HFNC and required intubation	In propensity-adjusted and -matched analysis, early intubation (<48 h) was associated with better overall ICU mortality (adjusted OR = 0.317, *P* = 0.005; matched OR = 0.369, *P* = 0.046)
	Messika et al. [55]	Prospective cohort	45 very severely hypoxemic patients with bilateral infiltrates who may be considered as ARDS patients	The intubation rate was 40 %. In the multivariate analysis, higher SAPS II scores were associated with HFNC failure
Cardiac surgery	Corley et al [10].	Prospective cohort	20 patients post-cardiac surgery. Impedance measures, P(aw), ratio, respiratory rate, and modified Borg scores were recorded first on COT and then on HFNC	HFNC significantly increased EELI. Tidal impedance variation, P(aw) and oxygenation, and reduced RR. HFNC also improved subjective dyspnea scoring
	Parke et al. [46]	Randomized	60 patients with mild to moderate hypoxemic ARF were randomized to receive HFNC or COT	HFNC patients tended to need NIV less frequently (10 % vs 30 %; *P* = 0.10) and had significantly fewer desaturations (*P* = 0.009)
	Parke et al. [47]	RCT	340 patients post-cardiac surgery who were randomized to receive either HFNC vs COT from extubation to day 2 after surgery	No differences in oxygenation on day 3 after surgery were observed, but did reduce the requirement for escalation of respiratory support (OR 0.47, 95 % CI 0.29–0.7, *P* = 0.001)
	Corley et al. [45]	RCT	155 patients with BMI ≥ 30 kg/m^2^. 74 patients received COT vs 81 patients treated with HFNC post-extubation	No difference was seen between groups in atelectasis. There was no difference in mean PaO_2_/F_I_O_2_ ratio or RR. Five patients failed allocated treatment in the control group compared with three in the treatment group (OR 0.53, 95 % CI 0.11–2.24, *P* = 0.40)
	Stéphan et al. [44]	Randomized non-inferiority controlled trial	830 patients who had undergone cardiothoracic surgery who developed ARF (failure of a spontaneous breathing trial or successful breathing trial but failed extubation) or were deemed at risk for respiratory failure after extubation due to preexisting risk factors. HFNC vs BiPAP	The treatment failed in 87 (21.0 %) of 414 patients with HFNC and 91 (21.9 %) of 416 patients with BiPAP (*P* = 0.003). No significant differences were found for ICU mortality (23 patients with BiPAP [5.5 %] and 28 with high-flow nasal oxygen therapy [6.8 %]; *P* = 0 .66)
Pre intubation	Miguel-Montanes et. al [43].	Prospective quasi-experimental before-after study	101 patients who were intubated. Non-inclusion criteria were age <18 years, intubation for cardiac arrest, severe hypoxemia (defined as SpO_2_ < 95 % with COT), patients already receiving HFNC, and patients under NIV	Median lowest SpO_2_ was 94 % with COT versus 100 % (95–100) with HFNC (*P* < 0.0001). Patients in the non-rebreathing bag reservoir facemask group experienced more episodes of severe hypoxemia (2 % vs 14 %, *P* = 0.03)
	Vourc’h et al. [42]	Randomized controlled trial	124 patients with PaO_2_/F_I_O_2_ ratio <300 mmHg, RR ≥30 bpm, and if they required F_I_O_2_ ≥ 0.5 to obtain a SpO_2_ of at least 90 %. Patients were randomized to HFNC or HFFM	No differences in the lowest saturation were observed (HFNC 91.5 % vs HFFM 89.5 %, *P* = 0.44). There was no difference for difficult intubation, ventilation-free days, intubation-related adverse events (including desaturation <80 %), or mortality
Post-extubation	Maggiore et al. [41]	RCT	105 patients with PaO_2_/F_I_O_2_ ≤ 300 before extubation who were randomized to 48 h of COT or HFNC	The PaO_2_/F_I_O_2_ was higher in the HFNC group (287 ± 74 vs 247 ± 81, *P* = 0.03). Comfort and airway dryness were also better with HFNC. HFNC patients had fewer interface displacements (32 % vs 56 %, *P* = 0.01) and oxygen desaturations (40 % vs 75 %; *P* < 0.001) and required reintubation (4 % vs 21 %, *P* = 0.01) or any form of ventilator support (7 % vs 35 %, *P* < 0.001) less frequently
	Rittayamai et al. [40]	Randomized crossover study	17 patients were randomized after extubation to receive either HFNC for 30 min followed by COT for another 30 min or COT for 30 min followed by HFNC for another 30 min	At the end of the study, patients with HFNC reported less dyspnea and lower RR and HR. Most of the subjects (88.2 %) preferred HFNC to COT
	Tiruvoipati et al. [39]	Randomized crossover study	50 patients were randomized to either HFNC followed by HFFM or HFFM followed by HFNC after a stabilization period of 30 min after extubation	There was no significant difference in gas exchange, RR, or hemodynamics. HFNC was better tolerated (*P* = 0.01) and tended to be more comfortable (*P* = 0.09)
Invasive procedures	Lucangelo et al. [38]	RCT	Patients were randomly assigned to three groups: 40 L/min through a Venturi mask (V40, N = 15), nasal cannula (N40, N = 15), and 60 L/min through a nasal cannula (N60, N = 15) during bronchoscopy	At the end of bronchoscopy, N60 presented higher PaO_2_/F_I_O_2_ and SpO_2_
	Simon et al. [37]	RCT	40 critically ill patients with hypoxaemic ARF to receive either NIV or HFNC during bronchoscopy in the ICU	The NIV group presented better oxygenation. Two patients with HFNC were unable to proceed to bronchoscopy due to progressive hypoxemia
Heart failure	Roca et al. [25]	Prospective cohort	Ten adult patients with New York Heart Association (NYHA) class III and left ventricle ejection fraction 45 % or less. Sequential echocardiographies were performed at baseline, using HFNC with 20 lpm and 40 lpm and post-HFNC	Median IVC inspiratory significantly (*P* < 0.05) decreased from baseline (37 %) to HFNC with 20 lpm (28 %) and HFNC with 40 lpm (21 %). Changes in the IVC inspiratory collapse were reversible after HFNC withdrawal
	Carratalá et al. [29]	Case series	Five patients with ACPE with stable dyspnea or hypoxemia following NIV	All patients were successfully treated with HFNC, presenting clinical and gasometrical improvements
ED	Lenglet et al. [36]	Prospective cohort	17 patients with ARF admitted to ED who required oxygen >9 L/min	HFNC was associated with better dyspnea scores. RR also decreased and oxygenation improved. Fewer patients with HFNC exhibited clinical signs of respiratory distress
	Rittayamai et al. [54]	Prospective randomized comparative study	40 hypoxemic patients were randomized to receive HFNC or conventional oxygen for 1 h	HFNC improved dyspnea and comfort. No serious adverse events related with HFNC were observed
Palliative care	Peters et al. [35]	Prospective cohort	50 DNI patients with ARF admitted to ICU	HFNC improved oxygenation and decreased RR

*APACHE* Acute Physiology and Chronic Health Evaluation, *ARDS* acute respiratory distress syndrome, *BiPAP* bilevel positive airway pressure, *BMI* body mass index, *bpm* breaths per minute, *CI* confidence interval, *COT* conventional oxygen therapy, *DNI* do not intubate, *ED* emergency department, *EELI* end expiratory lung impedance, *HFFM* High flow face mask, *HR* heart rate, *LOS* length of stay, *lpm* liters per min, *MV* mechanical ventilation, *NIV* non-invasive ventilation, *NNT* number needed to treat, *OR* odds ratio, *RCT* randomized controlled trial, *SOFA* Sequential Organ Failure Assessment, *SpO*_*2*_ pulse oximetry, *VFD* ventilator free days

### Acute hypoxemic respiratory failure

Its mechanisms of action and its clinical effects make the HFNC an attractive supportive tool for use in ARF patients. The first studies in ARF patients focused on physiological variables [16, 22, 23] and reported an improvement in oxygenation with reduced RR and no changes in PaCO_2_. Moreover, the HFNC was better tolerated and achieved a greater level of comfort than conventional oxygen devices [16, 23]. Although greater oxygenation improvements were obtained with NIV, the HFNC was usually better tolerated [31, 32]. In addition, HFNC has been used in small cohorts of patients with ARF in the emergency department [36, 54] and even in 150 children aged under 2 years during interhospital transport [56]. The effects on oxygenation and rates of intubation reported in all these studies are similar to those found when HFNC therapy has been used in ARF patients admitted to critical care areas, suggesting that, with adequate monitoring, HFNC therapy can be safely used outside critical care areas.

Moreover, early predictors of HFNC outcome have also been described. Sztrymf et al. [23] reported that RR as well as the percentage of patients exhibiting thoraco-abdominal asynchrony as early as 30 and 15 min after the beginning of HFNC therapy were significantly higher in patients who required endotracheal intubation. The PaO_2_/F_I_O_2_ ratio 1 h after the beginning of HFNC therapy was significantly lower in patients requiring invasive mechanical ventilation. Similarly, in a series of 20 H1N1 patients treated with HFNC, worse PaO_2_/F_I_O_2_ ratios were observed in patients who required intubation after 6 h of treatment [24]. However, in addition to respiratory variables, non-pulmonary severity may also be a good predictor of HFNC failure. Indeed, in two small cohort studies including H1N1 patients or lung transplant recipients requiring readmission to the ICU due to ARF, both treated with HFNC, the presence of shock has been associated with a higher risk of mechanical ventilation (MV) [24, 33]. Accurate, early predictors of HFNC therapy success are important, since a recent propensity-score analysis associated early intubation (within the first 48 h) with better ICU survival [53]. In spite of its limitations [57], the study by Kang et al. [53] raises an important issue: the fact that intubation should not be delayed in patients treated with HFNC, as delay may worsen their prognosis. Therefore, the ability to describe accurate predictors of HFNC therapy success which can allow timely endotracheal intubation in patients who are likely to fail is a point of special interest.

Another controversial issue is whether acute respiratory distress syndrome (ARDS) patients should be treated with HFNC. Moreover, it is unclear whether patients with bilateral infiltrates treated with HFNC could be considered as having ARDS. In fact, most of the patients included in studies have bilateral infiltrates [33, 34, 55]. The Berlin definition of ARDS [58] requires a minimum of 5 cmH_2_0 of positive end-expiratory pressure (PEEP) and it has been shown that HFNC can provide a level of PEEP which is higher at peak expiratory pressure [3, 5]. Moreover, ARDS does not begin at the time of MV onset. Therefore, it could be accepted that patients with a risk factor for ARDS, who are hypoxemic (PaO_2_/F_I_O_2_ ratio ≤300 mmHg) and have bilateral infiltrates not fully explained by cardiac failure or fluid overload, may be considered as ARDS patients [59]. In these patients, HFNC may achieve success rates [55] similar to those of NIV [60].

Besides the physiological improvement, one of the most important points is whether HFNC can reduce the need for further MV or mortality in ARF patients. The first results were reported in a randomized controlled trial (RCT) which included postoperative cardiac surgery patients with ARF [46]. HFNC patients were less likely to need escalation to NIV than those receiving conventional oxygen devices and also had fewer desaturations. In a recent retrospective analysis of a prospectively assessed cohort of 37 lung transplant patients readmitted to ICU due to ARF, HFNC therapy was the only variable at ICU admission associated with a decreased risk of MV in the multivariate analysis [33]. The absolute risk reduction for MV with HFNC was 29.8 % and only three patients needed to be treated with HFNC to prevent one intubation. Moreover, non-ventilated patients had an increased survival rate. More recently, the first large RCT to assess clinical outcomes with HFNC (50 L/min), conventional oxygen devices, and NIV has been published [34]. The study included 310 patients with hypoxemic ARF, defined as PaO_2_/F_I_O_2_ ratio ≤300 mmHg or RR >25 breaths per min (bpm) with 10 liters per min (lpm) of O_2_. Patients with a history of chronic respiratory disease, including chronic obstructive pulmonary disease (COPD), as well as patients with ACPE, severe neutropenia, and hypercapnia (PaCO_2_ > 45 mmHg) were excluded, as were patients with hemodynamic instability or on vasopressors at the time of inclusion. Moreover, patients in the NIV group were treated with NIV for a median of only 8 h during the first two days of randomization and received HFNC therapy between NIV sessions. The primary outcome—the rate of endotracheal intubation—did not differ significantly between the groups (38 % for the HFNC group vs 47 % for the conventional oxygen device group and 50 % for the NIV group, *P* = 0.18). This negative result may be due to the fact that the observed rate of intubation in the conventional oxygen devices was lower than expected and, therefore, the study may have been underpowered to identify differences in this endpoint. However, a post hoc adjusted analysis including the 238 patients with a PaO_2_/F_I_O_2_ ratio ≤200 mmHg found that HFNC reduced intubation rates (*P* = 0.009). In the entire cohort, HFNC therapy increased ventilator-free days, reduced 90-day mortality, and was associated with better comfort and lower dyspnea severity. In contrast, compared with HFNC patients, NIV patients presented higher 90-day mortality, probably due to the use of higher tidal volumes (Vt 9.2 ± 3.0 ml/kg).

### Invasive procedures

HFNC therapy has been used during invasive procedures such as bronchoscopy [37, 38, 61, 62] or endotracheal intubation [42, 43] both to prevent the appearance of hypoxemia and to treat hypoxemic patients. Regarding its use during bronchoscopy, although HFNC seem to be more useful than conventional oxygen devices in preventing and treating hypoxemia, NIV may be superior to HFNC in patients with severe hypoxemia [37]. HFNC may also be useful during transesophageal echocardiography or digestive tract endoscopy.

Two studies have evaluated the efficacy of HFNC therapy during endotracheal intubation [42, 43] and have produced conflicting results. First, in a prospective, quasi-experimental, before-after study, Miguel-Montanes et al. [43] assessed the usefulness of HFNC during tracheal intubation of critically ill patients with mild to moderate hypoxemia who were intubated mainly for reasons other than ARF compared with a non-rebreathing bag reservoir facemask. They observed that HFNC significantly improved preoxygenation and reduced the prevalence of severe hypoxemia. In contrast, Vourc’h et al. [42] did not report any differences in a more recent RCT comparing the effectiveness of HFNC with 15 L/min of oxygen delivered through a facial mask in preventing desaturation in patients with severe ARF, defined as a PaO_2_/F_I_O_2_ < 300, SpO_2_ < 90 % and a F_I_O_2_ ≥ 0.5 or RR >30 bpm. The differences in the two studies may be due to the differences in the type of patient included. However, the preoxygenation time of 4 min used in the study by Vourc’h et al. [42] may not have been long enough to reveal significant differences in oxygenation. Moreover, more patients in the HFNC group were treated with HFNC prior to inclusion (16.1 % vs 5.3 %), suggesting that the HFNC group may have been more hypoxemic before the start of the study.

### Post-extubation

Two preliminary physiological studies comparing HFNC with conventional oxygen devices using a crossover design and during a short period of time after extubation have confirmed the consistent benefit of HFNC in terms of overall comfort [39, 40] previously reported in ARF patients. Interestingly, however, both studies reported a significant improvement in quasi-objective surrogates of comfort, such as perceived dyspnea or tolerance score, when applied in a crossover design with conventional oxygen devices. Rittayamai et al. [40] observed a decrease in RR and heart rate when comparing HFNC therapy at 35 L/min vs conventional oxygen devices at 6–10 L/min in 17 patients during a 30-min period in a crossover study. In contrast, Tiruvoipati et al. [39] found no change in these physiological variables when comparing 30 L/min delivered through a HFNC or 15 L/min through high flow face mask.

Soon afterwards, the first large multicenter RCT comparing HFNC with conventional oxygen devices after extubation was published [41]. The study included patients with ARF due to pneumonia and trauma who were mechanically ventilated for a mean of almost five days before extubation. In these patients, the use of HFNC was associated with better comfort, better oxygenation, fewer desaturations and interface displacements, and a lower reintubation rate. However, the effectiveness of HFNC therapy during the extubation period in postoperative patients remains controversial; most studies on this issue have included patients after cardiothoracic surgery [44, 45, 47]. Parke et al. [47] included a non-selected population of cardiac surgery patients with mild to moderate ARF, observing that HFNC patients more frequently succeeded and could be weaned to conventional oxygen devices. In contrast, in patients randomized to conventional oxygen devices, ARF was more likely to worsen and escalation to NIV or HFNC required. Corley et al. [45] included a population of cardiac surgery patients with body mass index (BMI) ≥30 who were randomly assigned to prophylactic HFNC therapy or conventional oxygen devices after extubation. They did not observe any difference in atelectasis formation, oxygenation, respiratory rate, or dyspnea. Finally, the BiPOP study [44], a multicenter, non-inferiority RCT, compared HFNC and NIV for preventing or resolving ARF after cardiothoracic surgery. Three different types of patient were eligible: patients who failed after a spontaneous breathing trial; patients who succeeded but had a preexisting risk factor for postoperative ARF (BMI >30, left ventricular ejection fraction <40 %, and failure of previous extubation); and patients who succeeded after a spontaneous breathing trial but then failed extubation (defined as at least one of the following: PaO_2_/F_I_O_2_ < 300, RR >25 bpm for at least 2 h, and use of accessory respiratory muscles or paradoxical respiration). After randomizing more than 800 patients, HFNC therapy did not increase the rate of treatment failure (defined as reintubation, switch to the other study treatment, or premature treatment discontinuation at the patient’s request or due to an adverse event). Therefore, as HFNC therapy did not worsen outcomes, may be easier to administer, and requires lower nursing workload, the authors concluded that the results supported the use of HFNC in this subset of patients.

Certain questions remain unanswered, however, such as the optimal flow and the subset of patients who would benefit the most from HFNC therapy. Studies including other types of surgical patients, such as abdominal surgery (the OPERA trial) [63] and lung resection, will be published in the near future.

### Critically ill tracheostomized patients

Weaning of tracheostomized patients is still a challenge. To our knowledge, only one randomized trial has included high flow therapy in the protocol [64]. This was a single-center study including 181 critically ill tracheostomized patients who were randomized to have the tracheal cuff deflated or not during spontaneous breathing trials. All patients received high-flow conditioned oxygen therapy through a direct tracheostomy connection to the maximum tolerated flow and conditioned up to 37 °C. Although that study was not specifically designed to assess the effectiveness of high flow through the trachea, the authors hypothesized that HFNC therapy may have some benefits in the weaning process of tracheostomized patients with a deflated tracheal cuff. Positive airway pressure may theoretically reduce microaspirations and, with a deflated cuff, a higher flow is conveyed through the pericannular space, allowing for better drainage of secretions.

### Chronic diseases

#### Mucociliary clearance

COPD and bronchiectasis are both airway disorders characterized by neutrophilic airway inflammation, mucus hypersecretion and retention, and impaired mucociliary transport [65–70]. In these patients, some studies have also analyzed the effect of HFNC therapy on lung mucociliary clearance. In bronchiectasic patients, Hasani et al. [71] demonstrated that a period as short as 3 h/day of HFNC therapy at home over seven days significantly increased lung mucociliary clearance measured by radioaerosol labeling. Rea et al. [65] performed a 12-month randomized study with 108 patients diagnosed with COPD or bronchiectasis who received HFNC therapy ≥2 h per day in their home in order to examine the effects of HFNC therapy on the frequency of exacerbations, quality of life, lung function, exercise capacity, and airway inflammation. The results showed that patients on long-term HFNC therapy had significantly fewer exacerbation days, increased time to first exacerbation, and reduced exacerbation frequency compared with usual care. Quality of life scores and lung function at 3 and 12 months improved significantly with humidification therapy compared with usual care.

The preliminary results of a randomized, placebo-controlled, one-year study of 200 patients designed to evaluate the effect of HFNC therapy on patients in need of long-term oxygen therapy (86 COPD) have recently been reported [72]. The patients received between 20 and 30 L/min and, on average, HFNC therapy was used more than 7 h per day. Moreover, COPD patients treated with HFNC had fewer exacerbations and fewer hospital admissions than controls.

#### Sleep-related hypoventilation

Two short studies and one case-report have described the effects of HFNC therapy on nocturnal hypoventilation. Okuda et al. [73] reported a patient on HFNC therapy in whom the Apnea–Hypopnea Index decreased while oxygenation improved. HFNC therapy was continued at home without any recurrences of CO_2_ narcosis. Nilius et al. [74] assessed the effects of high flow nasal insufflations in 17 COPD patients with chronic hypercapnic respiratory failure, delivering a mixture of 20 L/min room air and 2 L/min O_2_ through a nasal cannula. High flow nasal insufflations led to a systematic reduction in RR without deterioration of the hypercapnia, suggesting that HFNC may improve efficiency of breathing and may be used as an adjunct to low flow oxygen for preventing hypercapnic respiratory failure in severely ill COPD patients. These same authors analyzed the effectiveness of HFNC therapy in 20 patients with severe COPD who were oxygen-dependent and had hypercapnic respiratory failure [75]. They found that, compared with oxygen alone, warm and humidified air at a rate of 20 L/min attenuated nocturnal hypoventilation in COPD patients with severe and hypercapnic respiratory failure.

#### Obstructive sleep apnea syndrome

The results of two short studies conducted in adult populations suggested that HFNC therapy could be an alternative to CPAP. The first study included 11 patients with mild to severe obstructive sleep apnea hypopnea syndrome [76]. In these patients, HFNC reduced the mean Apnea–Hypopnea Index and the respiratory arousal index. The second study analyzed the role of HFNC in ten patients with acute stroke and sleep-disordered breathing ranging from moderate to severe [77]. HFNC therapy was well tolerated and decreased the Apnea–Hypopnea Index and the oxygen desaturation index by >3 %. Moreover, the percentage of slow-wave sleep significantly increased and quality of sleep improved.

### Patients with a do-not-intubate order

In patients with a do-not-intubate order, it is mandatory to provide as high a level of comfort as possible. Even though NIV might be considered as a potential supportive measure, it is less likely to be tolerated than HFNC therapy [31]. A retrospective study analyzing the efficacy of HFNC therapy in do-not-intubate hypoxemic patients admitted to the ICU [35] found that it provided adequate oxygenation and comfort and that only 18 % of patients required escalation to NIV, thus suggesting that HFNC may be an alternative to NIV in do-not-intubate patients. Two more retrospective studies included patients with cancer [78] or hematological malignancies [79]. In this subset of patients, HFNC therapy should be considered as a way of enhancing comfort in patients with do-not-intubate orders and in those who do not tolerate conventional oxygen devices or NIV.

### Aerosol therapy

In patients with ARF, the use of aerosol therapy to treat bronchial hyperresponsiveness or pulmonary infections is becoming increasingly frequent. However, the efficiency of aerosol delivery with HFNC is likely to be low due to high flow rates, humidification, small delivery line diameters, and narrow flow passages in the cannula and abrupt changes in flow direction [80]. Therefore, questions that frequently arise are how to use aerosol therapy, where to implement the system, and whether it is effective.

Recently, an in vitro study analyzing albuterol (2.5 mg/3 mL) delivery using a vibrating mesh nebulizer (Aeroneb system, Aerogen Limited, Galway Business Park, Dangan, Galway, Ireland) during HFNC therapy was published [81]. The results showed that the amount of albuterol delivered was lower than the amount expected for a clinical response for the majority of flow rates and cannula size combinations. Other studies have shown that it is still possible to achieve a lung dose of 14 % even at flow rates of 30 L/min [82]. In this regard, other experimental studies have achieved significant improvements in delivery, with delivery efficiency up to 80 % at flow rates of 15 L/min [83]. Furthermore, HFNC therapy allows aerosol delivery without interruption of oxygen flow and pressure and it could be considered as an efficient delivery strategy for pulmonary aerosol medications [84].

Another interesting point to consider is the effect of the nebulizer position on aerosol drug deposition. The results of a recent in vitro study that simulated preterm infant settings showed that the mean percentage of dose delivered was greater with the nebulizer placed prior to the humidifier [80].

Interestingly, HFNC therapy can achieve stricter control of the administered F_I_O_2_ and may obtain totally conditioned gas during nebulization [85]. These two points are especially important in COPD patients.

We should note that all these studies have major limitations and that no strong evidence-based recommendations can be made. At very high flows, however, the amount of aerosol delivery is likely to be low. Moreover, the efficiency of aerosol delivery can be significantly improved by the use of moderate flows and it seems reasonable that if a vibrating mesh nebulizer is used in patients treated with a HFNC, it should be put in place prior to the humidifier.

## Recommendations for use in patients with ARF

Although many aspects of HFNC therapy remain unclear, evidence supporting its use in hypoxemic ARF patients is accumulating steadily. Here, we present our recommendations for applying HFNC therapy from the onset of ARF until weaning based on the current evidence and our experience. Like many other therapies used in critically ill patients, HFNC therapy should be started as early as possible. It seems logical to start with an F_I_O_2_ of 1 with the maximum tolerated flow up to 50 L/min [34]. The fraction of inspired oxygen should be subsequently titrated according to a target SpO_2_. The flow delivered will try to satisfy the inspiratory demand, minimizing the entrainment of room air and thus ensuring administration of the required F_I_O_2_. Although it seems reasonable to titrate the flow according to the comfort of the patient, we can hypothesize that if these very high flows increase tidal volume, they may generate some degree of alveolar overdistension, especially in patients with preexisting lung injury. Finally, if the patient progresses well and HFNC can be withdrawn, we first decrease the F_I_O_2_ and then, when the F_I_O_2_ is <0.5, we start to decrease flow. When F_I_O_2_ is <0.5 and the flow rate is <20 L/min, HFNC can be replaced by conventional oxygen devices. We stress that all patients with HFNC must be strictly monitored, paying special attention to oxygenation, RR, respiratory pattern, and need for vasopressors since all these variables have been implicated in HFNC failure. It is equally important not to delay intubation in patients who fail, as delay may worsen their prognosis.

HFNC therapy presents certain advantages over NIV. It is probably easier to apply, it needs less equipment, and it generates lower workloads. Importantly, it is more comfortable for the patient and is better tolerated and does not seem to be inferior in terms of clinical outcomes in patients with hypoxemic respiratory failure. However, HFNC therapy may not be indicated in all patients with ARF [86]. Currently, NIV should still be considered as a first-line treatment in a variety of clinical situations, such as hypercapnic respiratory failure [87] during COPD exacerbations [88] or ACPE [89]. In fact, as HFNC therapy generates only slight increases in airway pressure at end expiration, it is unlikely to reduce the work of breathing as effectively as NIV. Therefore, NIV appears to be more effective in patients who are more severely affected. Finally, at centers with substantial experience treating hypoxemic ARF with NIV, decisions on the strategy to use should be individualized.

## Future directions for research

Although evidence for the effectiveness of HFNC therapy is growing exponentially, several questions remain unanswered (Table 2). Future research should focus on identifying which patients will benefit the most from HFNC therapy. To achieve this, it might be worthwhile to perform RCT including patients with specific diseases rather than including all patients with different types of ARF, from pneumonia to ACPE to hypercapnic COPD. Selecting the right patients to treat and knowing how to use HFNC properly will improve results. In this regard, we need to identify and describe early predictors of HFNC success in order to minimize delays in intubations that could worsen patient outcomes. And it would also be desirable to establish the optimal flow rate to set up in each patient. While insufficient flow may lead to HFNC failure, excessively high flow may generate alveolar overdistension and increase the pre-existing lung injury. On the other hand, with strict patient monitoring to avoid delayed intubation in case of HFNC failure, HFNC can be used either in the emergency department [36, 54] or in hospital wards for the treatment of ARF patients [53]. However, we also need strong and robust cost-effectiveness analyses of HFNC therapy that support its use in different clinical situations. Finally, the evidence with patients with chronic respiratory diseases such as sleep-related hypoventilation, obstructive sleep apnea syndrome, or chronic cough is very limited [90]; what little is known is based on small series or case reports which are far from conclusive but which may generate hypotheses for further studies. Therefore, RCTs evaluating the efficiency of HFNC therapy in these situations are still mandatory.

**Table 2:** Areas of uncertainty and key questions in HFNC research

• RCT in specific etiologies of ARF
• Describing early predictors of HFNC success in patients with ARF
• Optimal flow rate titration
• Should hypoxemic ARF patients with bilateral infiltrates who are treated with a HFNC be considered as ARDS patients?
• Cost-effectiveness analysis
• RCT in chronic respiratory diseases

## Conclusion

Delivery of heated and humidified oxygen at high flow rates through nasal cannula is now widely used in adult patients. Its mechanisms of action and potential clinical benefits can help to improve the management of patients with either acute or chronic respiratory failure. With the evidence currently available, several questions still remain unanswered; in the absence of any general recommendations, decisions on HFNC treatment should be individualized in each particular situation. However, HFNC therapy is an innovative and powerful technique that is currently changing the management of patients with respiratory failure.

## Abbreviations

ACPE: acute cardiogenic pulmonary edema
ARDS: acute respiratory distress syndrome
ARF: acute respiratory failure
bpm: beats per min
CO_2_: carbon dioxide
COPD: chronic obstructive pulmonary disease
CPAP: continuous positive airway pressure
F_I_O_2_: fraction of inspired oxygen
HFNC: high flow nasal cannula
ICU: intensive care unit
IVC: inferior vena cava
lpm: liters per min
MV: mechanical ventilation
NIV: non-invasive ventilation
PaCO_2_: arterial pressure of carbon dioxide
PaO_2_: arterial pressure of oxygen
PEEP: positive end-expiratory pressure
RCT: randomized controlled trial
RR: respiratory rate
